# A Consideration of Some of the Responsibilities of Dentists and Physicians

**Published:** 1892-03

**Authors:** Lyman F. Bigelow


### A CONSIDERATION OF SOME OF THE RESPONSIBILITIES OF DENTISTS AND PHYSICIANS.[1]

BY LYMAN F. BIGELOW, D.M.D.

Just how far dentists and physicians are legally responsible for benefits conferred and injuries done to patients by failing to do all that should be done, or by doing for them operations based on unsound judgment and unskilfully performed, is something we, as members of one profession, are not allowed to decide for ourselves, but are at times forced to meet members of another profession and

---

[1] Read before the Harvard Odontological Society, June 25, 1891.

be passed upon by them. Though justice is not always obtained by such procedure, the very fact that protection may be sought from an outside source has, I believe, a restraining influence on the more powerful classes.

The tremendous advantage the professional man has over his clients, be he dentist, physician, or lawyer, is, it seems to me, unequalled by the advantage a man of any mercantile calling has over his customers. It is true that syndicates and trusts are formed, and prices, it sometimes seems to appear, are unjustly inflated. Yet the alternatives for the consumer are much greater than those of the person who places himself in our care for advice and assistance. The people who confidentially disclose their aches and pains to the dentist and physician are placing themselves in very intimate relation with their advisers; and we as advisers are in a very responsible position.

We receive patients for the first time, and from the moment we begin to make an examination to the final service rendered with subsequent advice we are in a most responsible relation to the patient, and this does not cease at the close of a given set of operations. We are in a measure accountable for the future comfort and pleasure of our patients so far as comes within our province. It is perfectly easy to conceive of a case where, by a poor appreciation of existing conditions and poor judgment of the possibilities, we may make one's future full of unpleasantness without the opportunity of ever making their condition as satisfactory as it might have been had we been better able to judge and advise. We all see cases which seem to show a lack of just the qualities I have intimated we should possess.

We are responsible morally so far as we have capacity to acquire knowledge and skill. It is not possible for some of us to weigh as accurately as others the case in all its bearings, past, present, and future, yet we do it with as much conscientiousness as though we had all knowledge, and morally we are doing our duty; but, as has already been said, we are not permitted to determine for ourselves what we shall or shall not do from the legal stand-point, and, as we all know, members of our profession have been obliged to defend themselves in court against serious charges, perhaps just, perhaps unjust.

From Wharton and Stillé's "Medical Jurisprudence" I quote the following: "According to Casper, malpractice is imputable in cases where the practice adopted is in conflict with general rules prescribed for similar cases by contemporary medical science, and adopted in contemporary medical experience. By Saxinger this definition is objected to because it cannot be applied in many cases of malpractice, and because there are no general rules applicable to every case. The physician, he argues, would be compelled, if this standard were made obligatory, to surrender all independent activity. But in any view it must appear to sustain process against a physician for malpractice to show, first, that the injury to the health or body resulted from bad treatment of the case by the physician; second, that this evil result might have been foreseen and avoided by a competent practitioner, and, further, dentists are subject to the same conditions of liability as are other practitioners, and the patient cannot recover for a mistake into which he himself led the dentist; that the service was gratuitous is no defence to a suit for negligence."

You have heard the quotations from a legal authority concerning the responsibilities of physicians and dentists, yet it seems hardly safe to allow that the general rules governing the practice of many men of our profession are safe rules for those to follow who have at heart the greatest good for their patients.

For instance, we often hear (perhaps not in our offices, but outside) people say that their dentist or physician would not extract a tooth until the inflammation was reduced, or that no treatment could be given teeth in such condition that would get them into a serviceable and comfortable state, therefore, recommend extraction. Is not this quite a general practice? But, in the first case nature is trying to throw off an irritating substance, and if extraction is indicated by other conditions, it is, as a rule, just the proper time to extract; while in the second case we are all familiar with teeth that have been, for a long time, in the condition of Vesuvius, smouldering within, only waiting for right conditions to prevail, when they will throw off the products of that fire; even where eruptions have taken place at very frequent intervals, we have been able to control conditions, put the member in a comparatively passive and useful condition, and avoid extraction. It therefore seems we are justified in violating some of what seem to be general rules of practice of many contemporary with our own time.

In the country, physicians are often called upon to relieve pain arising from a diseased condition of the teeth; and from reports that are made, it would seem that their appreciation of the value of these organs is not what it should be to properly fill the requirements of a general practitioner. They seem to forget, too, that there are those to whom patients of this class might be referred who have made diseases of these organs a special study, and are better able to prescribe than they. How often do we hear of physicians giving long courses of treatment for neuralgia that short operations of the dentist have seemed to entirely cure; not that the dentist has marvellous power, but that the physician failed utterly in his diagnosis, or possibly that he did not care to acknowledge that another might be able to do what he could not.

I wish to cite two cases, not to show that in either were the parties, according to common law, responsible, but to bring to your attention the fact that physicians and dentists, as a rule, are too poorly qualified to deal with conditions that are constantly being presented, and that dentists, especially we younger members, should be extremely careful in making recommendations and performing operations, that we may not do serious injury to our patients, nor reflect unpleasantly on our professional brothers.

First, that of a young lady, about twenty-one years of age, who had been a patient of a well known dentist of Boston, but it being quite inconvenient for her to reach him, she consulted another, and received treatment from him which she has described in the main as follows: He examined her teeth and recommended the removal of the pulps of the left superior first bicuspid and right inferior first molar, and that it might be necessary to do the same with both superior first molars; that both superior second bicuspids be extracted, giving as his reason that it would be necessary for him to do this, as the teeth were in a badly diseased condition, saying also that he could not get at the molars next to them to fill unless the bicuspids were removed. He diagnosticated the condition of the teeth that were to have their pulps removed, and those that were to be sacrificed by extraction by grasping them between his thumb and forefinger and moving them from side to side, claiming that the movement was sufficient to indicate a badly diseased condition at the apex of the roots. He extracted the teeth condemned and removed the pulps from those mentioned as requiring such operations, treated and filled them. So far as I have been able, from close questioning of the patient and an examination of her mouth and teeth soon after the operations were performed, as well as an examination of the teeth that were extracted (they being brought to me by the dentist himself), I could not possibly imagine such a diseased condition of the teeth as he personally told me did exist and yet find her mouth so exceptionally good so soon after.

The teeth were extracted at the first sitting, and the indignation of the young lady's parents was very great, they and she preferring to undergo almost any treatment rather than lose a tooth by extraction.

During the process of pulp-removing and filling, which was heroically done, he making no application to destroy the life of the pulps, the patient suffered a great deal, being unable to sleep on the nights following the removal of the pulps, and was in an exceedingly nervous condition. The dentist told her she must endure the pain, and also said she would not have as much if she remained in the house evenings and did not dance so much, with other equally strange things. Fortunately or unfortunately for me, she came for treatment at the suggestion of her sister and mother, I, however, telling her that I thought she had better rest awhile before undergoing any more dental operations, thinking that her trouble might be imaginary. When she came to me she described trouble in the bicuspid that had been treated. Upon removal of the filling in the crown and pulp-chamber I found the root unfilled. Ventilation and the treatment given have made it comfortable. I have recently been treating the molar that was devitalized and left in a similar condition, and have given some relief.

Each one of us has his own particular methods of manipulation. These we have a perfect right to. We have individual discriminating power. This we are heir to, and we can cultivate it and perfect it in a great or small measure, and the more it is cultivated the greater do the responsibilities and possibilities become. In the case cited there was seemingly a lack of this power, or (which I do not like to think) that advice was given and operations performed without the best interest of the patient at heart. It is a peculiar case in all its bearings, and the conversations had with the dentist make it appear even more so, it being impossible for me to dissect his language sufficiently well to get at just what he did mean.

The second case I wish to cite is that of a clergyman who applied to a physician in the country for relief of aching teeth, he locating the origin of his trouble in the right superior bicuspid region.

He had been kept awake several nights, and was becoming very much exhausted. The physician made a slight examination of his mouth, and finally extracted the tooth indicated by the patient, who wished him to remove it. His troubles were not in the least abated, but, on the contrary, from disappointment at not getting relief and the soreness of the wound thus made, they were multiplied.

Very soon after, I saw him and located the primary cause of his suffering in the right inferior third molar, it having an exposed pulp. The instant I touched the pulp, he declared it to be just where the pain was, though, before looking into his mouth, he described to me the ache as being in the right superior bicuspid region. He was greatly surprised, but you are not, and I was not, for we all have often seen similar cases. I made an application that relieved him almost immediately, and subsequently treated and filled the tooth.

From these two cases, that have very recently come under my observation, I have learned a very valuable lesson, and the responsibilities of our calling were brought vividly to my mind. In the first, that of the young lady with ample means at command and a disposition to have her teeth and mouth kept in as good condition as possible, ready to make and keep appointments with the dentist to whom she applies, with all confidence in his ability and judgment, surrendering herself to him with the understanding that he is to do for her just what should be done for her best good, the loss of these pulps and the removal of the two bicuspids that she declared had given her no trouble is a serious matter with her.

In the case of the clergyman, who was anxious to rid himself of suffering even at the expense of a tooth, if it were necessary to do so, imagine his vexation when his troubles continued after the loss of the tooth he supposed was causing the pain. A tooth to a man with such prospects in life as has this one is of infinite value; one whose calling is such that he is constantly before exacting audiences and whose every intonation is as sharply watched as to its merits as what he may say, feels the loss greatly, especially as it seemed to be utterly unnecessary. And yet, according to the authority quoted, there is no redress for him, it being true that he led the physician to make the mistake.

Were I to suggest any way to protect the professional man as well as his patients, it would not be in the nature of enacting more stringent laws regarding the penalty for misjudgment, but it would be, possibly, to require those intending to practise general medicine to acquire much more knowledge of the diseases of the mouth and teeth; possibly even better, to suggest that physicians confine themselves to relieving pain of aching teeth by other means than extraction, and referring such patients to the specialist.

So far as dentists, as a rule, are concerned, we should possess a much broader general education, a very much deeper knowledge of our specialty, and a good knowledge of general medicine, not that we are to practise it as such, but that we may grapple with cases that come within our province, and which are sufficiently numerous and perplexing, with a firmer and more accurate conception.

Institutions that profess to give young men this special professional education should, I believe, require not only a fair general education, but a liberal one, and, before graduating and assuming the responsibilities they must later face, students should undergo a much more comprehensive examination.

